# *Metarhizium*-Inoculated Coffee Seeds Promote Plant Growth and Biocontrol of Coffee Leaf Miner

**DOI:** 10.3390/microorganisms12091845

**Published:** 2024-09-06

**Authors:** Jéssica Letícia Abreu Martins, Mayara Loss Franzin, Douglas da Silva Ferreira, Larissa Cristina Rocha Magina, Elem Fialho Martins, Laís Viana Paes Mendonça, Wânia dos Santos Neves, Angelo Pallini, Fernando Hercos Valicente, Jason M. Schmidt, Simon Luke Elliot, Madelaine Venzon

**Affiliations:** 1 Department of Entomology, Federal University of Viçosa, Viçosa 36570-900, Brazil; jessica.abreu@ufv.br (J.L.A.M.); douglas.s.ferreira@ufv.br (D.d.S.F.); lais.v.mendonca@ufv.br (L.V.P.M.); pallini@ufv.br (A.P.); selliot@ufv.br (S.L.E.); 2 Nestlé Enterprise, São Paulo 04730-090, Brazil; 3 Department of Biology, Federal University of Viçosa, Viçosa 36570-900, Brazil; 4 Agriculture and Livestock Research Enterprise of Minas Gerais (EPAMIG), Viçosa 36570-000, Brazilwaniaepamig@yahoo.com.br (W.d.S.N.); 5 Embrapa Maize & Sorghum, Sete Lagoas 35701-970, Brazil; fernando.valicente@embrapa.br; 6 Department of Entomology, University of Georgia, Tifton, GA 31793 0000, USA

**Keywords:** sustainable pest management, biological control, *Leucoptera coffeella*

## Abstract

*Metarhizium* (Hypocreales: Clavicipitaceae) has a multifunctional life cycle, establishing as a plant endophyte and acting as entomopathogenic fungi. *Metarhizium robertsii* and *Metarhizium brunneum* can be associated with coffee plants and provide enhanced protection against a major pest of coffee, the coffee leaf miner (CLM) (*Leucoptera coffeella*). This association would be an easily deployable biological control option. Here we tested the potential of inoculating coffee seeds with *M. robertsii* and *M. brunneum* collected from the soil of coffee crops in the Cerrado (Brazil) for control of the CLM and the enhancement of plant growth with a commonly used fungicide. We conducted the experiment in a greenhouse and after the seedlings grew, we placed them in a cage with two couples of CLMs. We evaluated the CLM development time, reproduction, and plant growth traits. We observed a longer development time of CLMs when fed on plants inoculated with both isolates. In addition, the CLMs laid fewer eggs compared to those fed on plants without fungal inoculation. Plant growth was promoted when seeds were inoculated with fungi, and the fungicide did not affect any evaluated parameter. Coffee seed inoculation with *M. robertsii* and *M. brunneum* appears to provide protection against CLMs and promote growth improvement.

## 1. Introduction

Entomopathogenic fungi are important allies in the biological control of crop pests [1], and, besides their efficiency, they have a lower contamination risk associated with sprays [2], pest selectivity [3], and feasibility of being acquired in low-cost substrates for mass production by companies and farmers [4]. In this way, financial investments fostered by public and private initiatives for the development of new products based on fungi are promising, and initiatives for implementation are growing worldwide [5].

There is currently a diversity of entomopathogenic fungi available for pest control, and three genera are presently the most used for plant protection: *Beauveria* (Hypocreales: Cordycipitaceae), *Cordyceps* (Hypocreales: Cordycipitaceae) and *Metarhizium* (Hypocreales: Clavicipitaceae) [1,6,7]. In particular, *Metarhizium* is reported to control many insect pests across multiple agricultural systems including sugarcane, soybean, corn, and coffee crops [1,8,9,10]. Studies suggest its compatibility with pesticides and specificity to common pests [11,12,13,14]. The *Metarhizium* genus is also known for having a multifunctional life cycle. It acts as an entomopathogenic fungi and is capable of establishing in the rhizosphere of plants or engaging in endophytic relationships with plants [15,16,17,18]. The mutualist interactions of *Metarhizium* and plants can promote plant nutrient uptake and induce plant defenses to abiotic and biotic stresses [19,20,21,22].

The coffee leaf miner (CLM) or *Leucoptera coffeella* (Guérin-Mèneville) (Lepidoptera: Lyonetiidae), is one of the most important pests in coffee crops in Brazil [23]. The CLM significantly damages leaves and reduces photosynthetic rates, which drastically decreases coffee productivity [24]. Unfortunately, CLM control is mainly reliant on an array of pesticides that are not always successful and have led to pest resistance and resurgence [25,26,27]. Moreover, coffee produced under intensive chemical pest control can lead to violations of acceptable levels of pesticide residues allowed in the international market [28]. Encouragingly, recent work provides evidence that biocontrol and plant growth can be enhanced with root drenches of *Metarhizium* because these fungi can associate with coffee roots, which promotes plant growth, resistance to water stress, and protects against CLMs [9,29]. 

Ideally, endophytic associations that can be established early in the development of coffee plants and provide extended control could promote biological control throughout the growth from seed to production plants. Here, we hypothesize that inoculating coffee seeds with *Metarhizium* will generate seedlings with accelerated growth and enhanced protection against CLMs. We tested this hypothesis using two field-collected isolates of *Metarhizium brunneum* Petch [30] and *Metarhizium robertsii* [31] both previously recovered from the soil by Franzin et al. [9]. We also tested whether this new inoculation methodology, in conjunction with the use of fungicide, would be viable for (i) protecting the plant against CLM; (ii) promoting vegetative growth; and (iii) reducing the number of insects from the CLM second-generation.

## 2. Materials and Methods

### 2.1. Fungal Isolates and Suspensions for Seed Inoculations

We selected two isolates from the Agriculture and Livestock Research Enterprise of Minas Gerais isolate bank (EPAMIG Sudeste), *M. robertsii* RD-20.114 and *M. brunneum* RD-20.120. These were originally obtained from the *Coffee arabica* variety “IAC 44” roots in 2020, collected in the Cerrado’s savanna-like biome (18°9′48″ S and 46°59′00″ W) using a bait system of *Tenebrio molitor* larvae (Coleoptera; see Franzin et al. 2022 [9]). We inoculated the fungi on Petri dishes with potato dextrose agar (PDA) culture medium plus 0.05 g of chloramphenicol. The dishes were placed in an incubator in darkness at 26 °C for 14 days. By the end of this period, the fungi had sporulated and were ready for multiplication in a solid substrate. For this, we autoclaved 100 g of type 1 rice with 30 mL of distilled water for 20 min. After cooling, we placed the *Metarhizium* conidia obtained from three Petri dishes the size of 9 × 15 mm into each plastic bag with rice and incubated them at 26 °C for 14 days, by which time the fungi had sporulated on the rice grains and could be used to make spore suspensions.

To make spore suspensions, we added 50 g of rice grains with sporulating fungus in 1 L of sterile Tween solution at 0.05%. We added the Tween solution and stirred the mixture via inversion for 1 min to disperse the grains in the liquid. We filtered this through sterile gauze to remove rice grains and hyphal fragments. The suspension obtained was vortexed for 30 s. We adjusted the concentrations to 1.0 × 10^8^ conidia with the aid of a Neubauer chamber. To assess conidial germination, we transferred 150 μL of the suspension onto Petri dishes (9 cm diameter) with PDA + chloramphenicol (0.05 g L^−^^1^) and incubated these at 26 °C for 24 h. We then count the germinated conidia in a 100× stereomicroscope; we consider them to have germinated when their germ tube had grown at least twice as long as the conidia diameter.

### 2.2. Coffee Seed Inoculation

We selected 60 *C. arabica* seeds of the Catuaí Vermelho 144 variety. From these, to represent our control groups, 30 seeds were not treated with any fungicide and another 30 were treated with Tecto SC^®^ (active ingredient thiabendazole) at a concentration of 1 mL kg^−1^ of seed. Thus, we used 10 seeds in each proposed treatment. The fungicide-treated seeds were commercially obtained with the fungicide application carried out by the seller. The use of this fungicide is normally carried out to increase the shelf life of coffee seeds that are stored in cold chambers. Before being obtained, those seeds were stored in a cold chamber for about 45 days. Before the inoculation, we sterilized all the seeds in 0.5% sodium hypochlorite solution and 70% ethanol for 2 min. After that, all 60 seeds were submerged in a 100 mL suspension of conidia of either *M. robertsii* or *M. brunneum* at concentrations of 1.0 × 10^8^ conidia mL^−1^. We kept the seeds in this suspension for two hours, following the methodology of Canassa et al. [32].

### 2.3. Coffee Seedling Cultivation

We sowed the seeds in polyethylene plastic bags (20 × 30 × 20 cm) filled with commercial substrate and kept these in a greenhouse. During cultivation, we irrigated the plants according to their water needs. We did not use any pesticides at any time during the experiment, and we fertilized the plants monthly with 10 mL of 4 g L^−1^ ammonium sulfate per seedling. For 240 days, we maintained plants inside wooden gauze-sided cages (60 × 30 cm) to avoid insect infestations. The development time of the seedlings was prolonged due to the low temperatures recorded during the growing time. They were below 18 °C for about 100 days, between May 2021 and January 2022, when normally temperatures range from 18 °C to 23 °C. At the latest, the time required between seed germination and the formation of the seedlings is about 180 days [9].

### 2.4. Insect Rearing

For CLM rearing, we collected CLM-infested coffee leaves from experimental fields of the Diogo Alves de Mello Experimental Station at the Federal University of Viçosa, Viçosa, state of Minas Gerais, Brazil (20°45′14” S; 42°52′55” W). Insect rearing was conducted at the Biological Control laboratory of EPAMIG Sudeste following the methodology of Martins et al. [33]. We kept mined leaves inside acrylic cages (40 × 40 × 40 cm), and to keep the leaves turgid, petioles were immersed in sterile sponges in plastic containers (20 × 20 cm) of water and covered with polyurethane foam. As the insects emerged, we transferred them to new cages with clean coffee plants, allowing the insect life cycle to continue.

### 2.5. Effect of M. robertsii and M. brunneum Inoculation on CLM Development

We conducted the experiment in a greenhouse and each treatment had 10 replicate coffee seedlings. We established the following treatments: C1 (untreated seeds); C2 (fungicide-treated seeds); T1 (untreated fungicide seeds plus *M. robertsii*); T2 (fungicide-treated seeds plus *M. robertsii*); T3 (untreated fungicide seeds plus *M. brunneum*); and T4 (fungicide-treated seeds plus *M. brunneum*). The potted seedlings with two pairs of leaves, originated from seeds that had been inoculated as described above, were kept in cages measuring 60 × 30 cm with metal rods on the side and gauze on the faces [9]. In each cage we added a newly emerged CLM couple obtained from the CLM laboratory rearing and left them for 48 h. Next, we removed the adult CLM couples and counted, with the aid of a magnifying glass, the number of eggs the females laid on each seedling. From these eggs, we began our daily evaluations of every single CLM development in the plants inside of the cages (i.e., time from egg to adult) and continued until all the adults from the laid eggs had emerged. After the evaluations, we checked for the presence of fungi in all plants and plant parts. We placed leaf, steam, and root fragments in a potato dextrose agar (PDA) culture medium. 

### 2.6. Effect of M. robertsii and M. brunneum Inoculation on CLM Second-Generation

To verify the effects of *M. robertsii* and *M. brunneum* on the CLM second-generation, we collected 15 adult couples from each of the previous treatments, including the control. After that, we placed each couple into a 500 mL plastic container, along with a new coffee leaf with its petiole inserted in a 50 mL plastic container of water to maintain leaf turgor until the end of the evaluations. We evaluated the survival times of the males and females and the number of daily deposited eggs until both adult insects were dead.

### 2.7. Effect of M. robertsii and M. brunneum Inoculation on Plant Development

After the period of CLM evaluations, we removed all plants from the pots and cleaned the roots with a brush. With a measuring tape, we measured the shoot and root system lengths. For the shoot, we measured the distance from the first secondary root to the apical region of the plant. For the root, we measured from the first secondary root down to the root cap. After that, we cut the shoot and root systems and weighed their fresh and dry masses with a precision scale. Their dry weight was obtained after 24 h in a drying oven at 65 °C.

### 2.8. Statistical Analysis

We used the response variables including the number of laid eggs, the time of CLM development, the survival of emerged adults, and the plant parameters (i.e., root and shoot length, root and fresh dry weight) to investigate whether the two species of *Metarhizium* and their combination with fungicide affected the growth of coffee plants and CLM survival and reproduction. We analyzed the insect development times and the survival of the emerged males and females using ANOVA and survival analyses with censored Weibull distributions.

Data on the number of eggs from the second generation of CLM were analyzed with generalized linear mixed models (GLM) adjusted to a Poisson distribution. To analyze the CLM number of eggs we used ANOVA tests and pairwise comparisons with the emmeans package (v 1.10.4) [34]. For plant development parameters we used an analysis of deviance (F-tests) assuming a normal distribution. In case of significant results, pairwise comparisons were performed with the emmeans R-package (v 1.10.4) (adjustment method: Tukey) [35]. For the analyses, we considered the plants that had fungi in the tissue, and we found them in all the plants used in the experiments, except in the control ones.

## 3. Results

### 3.1. Fungi Recover from Plants and Identification

We recovered the fungi from all the plants and we found *Metarhizium* in all the plant roots, except the control ones. We performed visual identification of the fungi based on the structure of their conidia, thus validating the inoculation and persistence of the fungi in the plants. We cut 2 mm root fragments, performed superficial sterilization through immersing them in 70% alcohol and 5% sodium hypochlorite, then rinsed them in distilled water and dried them on sterile filter paper. We then placed them on Petri dishes with a potato dextrose agar medium to allow the growth and development of the endophytic fungi present in the plants. After the fungal structures grew, we identified them under a microscope at 40× magnification (Figure S1).

### 3.2. Effect of M. robertsii and M. brunneum Inoculation on CLM Development

**Figure 1:**
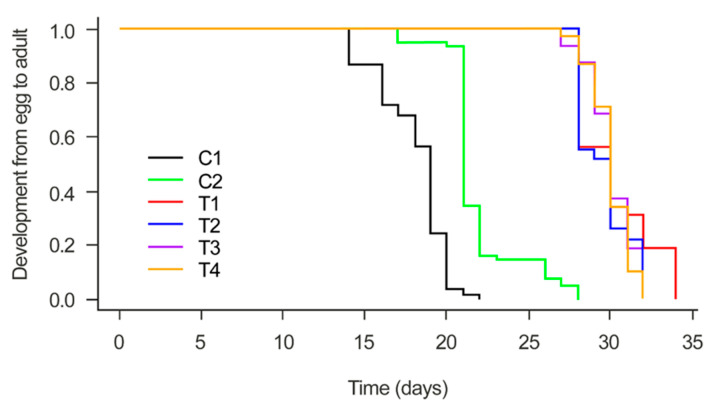
Developmental time from egg to adult of *Leucoptera coffeella* from coffee seeds inoculated with the treatments: C1 (untreated seeds); C2 (fungicide-treated seeds); T1 (untreated seeds plus *M. robertsii*); T2 (fungicide-treated seeds plus *M. robertsii*); T3 (untreated seeds plus *M. brunneum*); and T4 (fungicide-treated seeds plus *M. brunneum*).

When evaluating the total duration of the CLM life cycle (i.e., from egg to adult) in response to *Metarhizium* seed treatments, we observed that both isolates caused an increase in the total time of the CLM life cycle, when compared to the controls (X^2^ = 404.32, *p* < 0.05) (Figure 1).

### 3.3. Effect of M. robertsii and M. brunneum Inoculation on CLM Second-Generation

**Figure 2:**
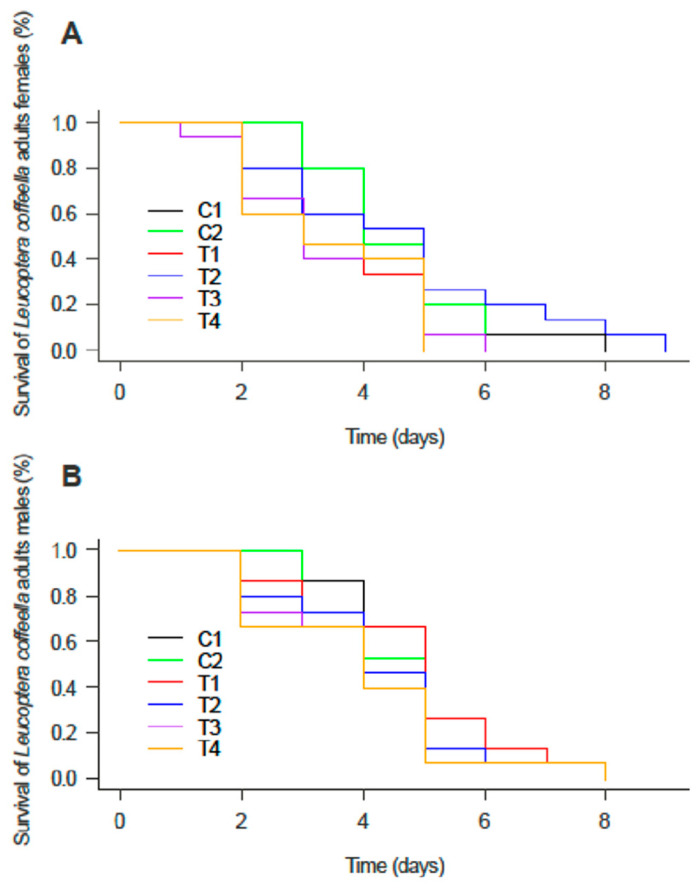
Survival of adult females (**A**) and males (**B**) of *Leucoptera coffeella* when fed on coffee plants following the treatments: C1 (untreated seeds); C2 (fungicide-treated seeds); T1 (untreated seeds plus *M. robertsii*); T2 (fungicide-treated seeds plus *M. robertsii*); T3 (untreated seeds plus *M. brunneum*); and T4 (fungicide-treated seeds plus *M. brunneum*).

**Figure 3:**
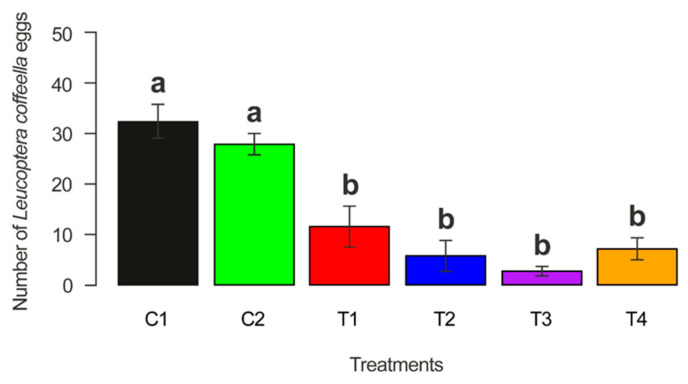
Number of eggs per female of *Leucoptera coffeella* that emerged from coffee seedlings grown from seeds inoculated with the treatments: C1 (untreated seeds); C2 (fungicide-treated seeds); T1 (untreated seeds plus *M. robertsii*); T2 (fungicide-treated seeds plus *M. robertsii*); T3 (untreated seeds plus *M. brunneum*); and T4 (fungicide-treated seeds plus *M. brunneum*). Bars with the same letters are not statistically different via the Tukey method (*p* < 0.001).

The inoculation of the coffee seeds with the *Metarhizium* isolates did not affect the survival of second-generation males and females (X^2^ = 0.61378, *p* = 0.9874) (Figure 2A), which did not differ from the controls (X^2^ = 1.3255, *p* = 0.9323) (Figure 2B). Regarding the total number of eggs laid per female, a significantly lower oviposition was found for females that emerged from plants, whose seeds were treated with the fungi compared to the controls (X^2^ = 786.77, *p* < 0.05) (Figure 3). Furthermore, the presence of the fungicide was also unable to affect the development of CLM males and females (Tables S2 and S3).

### 3.4. Effect of M. robertsii and M. brunneum Inoculation on Plant Development

**Figure 4:**
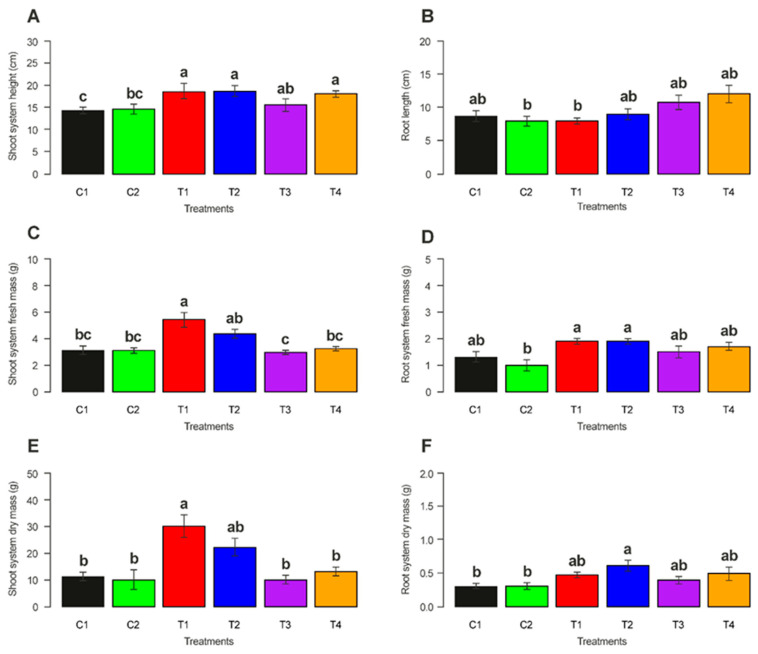
Growth variables of plants from coffee seeds: C1 (untreated seeds); C2 (fungicide-treated seeds); T1 (untreated seeds plus *M. robertsii*); T2 (fungicide-treated seeds plus *M. robertsii*); T3 (untreated seeds plus *M. brunneum*); and T4 (fungicide-treated seeds plus *M. brunneum*). (**A**) root length; (**B**) shoot system height; (**C**) shoot system fresh mass; (**D**) root system fresh mass; (**E**) root system dry mass; and (**F**) shoot system dry mass. Bars with the same letters are not statistically different via the Tukey method (*p* < 0.001).

The treatment of coffee seeds with the two isolates increased the height of the plant shoot system (Figure 4A) (F = 13.133, *p* = 0.005). The plants from the fungicide-treated seeds had longer roots (Figure 4B) (F = 14.359 *p* < 0.001), heavier fresh mass shoots (Figure 4C) (F= 12.470, *p* < 0.001), heavier root fresh mass (Figure 4D) (F = 4.2923, *p* = 0.0021), heavier shoot dry mass (Figure 4E) (F = 19.287, *p* < 0.001) and heavier root dry mass (Figure 4F) (F = 1.7818, *p* = 0.0007) compared to the control treatments.

## 4. Discussion

The isolates *M. robertsii* and *M. brunneum* used in our experiments as coffee seed inoculants lengthened the development time of the CLM adults. We also found a reduction in the number of eggs obtained from the CLM females developed on plants from fungal-inoculated seeds, which suggests fungal effects on subsequent generations of the pest. One of the possible explanations for the impaired development and reproduction of CLMs is the presence of destruxins, which are peptides produced by the secondary metabolism in some entomopathogenic fungi [36]. The action of destruxins synthesized by *M. robertsii* was previously linked to causing food poisoning in herbivorous insects and herbivore repellency [37]. In the case of *M. brunneum*, the presence of destruxins has also been reported to suppress insect immunity, making them more susceptible to entomopathogens [38]. Thus, it is possible that the destruxins reduced foliar consumption by the herbivores and indirectly affected their reproductive performance, an effect reported by Ahmad et al. [15] for *Agrotis ipsilon*. In addition to destruxins, some secondary fungal compounds such as saponins, terpenoids, and phenolic acids may have contributed to an anti-feeding effect on the CLMs, as demonstrated in other herbivores [19,39].

Our results suggest that *M. robertsii* and *M. brunneum* promoted an increase in dry and fresh mass of the shoot system in coffee plants. Therefore, the species can be used as growth promoters, possibly due to the increase in nutrient absorption through the roots, which can influence the health status of the plant and thereby its resistance to pest attacks [40]. Another interesting point demonstrated by Schenkel et al. [41] is that the mutualism between fungi and the host plant can modulate the emission of some plant volatile compounds. This alteration in volatile emissions can affect the choice of insect pests, the architecture of their root system, and even the ability of these fungi to manipulate insect–plant interactions. In our studies, we were not able to test this effect, but future works will test the effectiveness of *Metarhizium* in this choice process on coffee systems.

Besides the endophyte relation with plants and its negative effect on herbivores, the presence of *Metarhizium* in the rhizosphere of the plant can also contribute to pest management [42,43]. One application of this effect was studied by Franzin et al. [9]. They tested the same isolates used in this work and revealed that when the fungi were applied via drenching the soil, CLMs developed slower, with a lower percentage of mined leaf area on leaves, and in the case of *M. robertsii*, the number of adults per coffee seedling and the number of eggs of the progeny were reduced. Therefore, *Metarhizium* fungal associations with coffee roots are able to negatively affect CLMs and promote plant growth parameters. Our studies show that inoculation can successfully be accomplished at the seed stage and has the potential to facilitate a cost-effective inoculation process for the producers.

Coffee seeds are frequently treated with a post-harvest fungicide and dried to prepare for seedling production and to protect them during storage. Our experiments were also designed to assess the compatibility of *Metarhizium* seed inoculation with the commonly used thiabendazole-based fungicide seed treatment. Promisingly, thiabendazole presence apparently did not inhibit the development of *M. robertsii* or *M. brunneum*. One of the factors that may explain this absence of negative effects on the development of the entomopathogenic fungus may be linked to the short safety interval registered for seed treatment, which is two days. The thiabendazole is registered in Brazil to control fungi of the genus *Colletrotrichum* (Phyllachorales), *Penicillium* (Eurotiomycetidae), and *Fusarium* (Hypocreales) [44]. Although *Fusarium* sp. belongs to the same order as the genus *Metarhizium*, the period between seed fungicide application and the inoculation with entomopathogenic fungi (i.e., approximately 45 days) was sufficient for the fungicide residues to degrade, and *Metarhizium* was unaffected. Another favorable factor for the persistence of *Metarhizium* in the soil may also be associated with its saprophytic capacity (i.e., ability to survive in dead plant or animal material), which would help with its maintenance for so long in the soil [45,46].

Fungi inoculation of seeds to enhance crop protection is reported for other plant species, such as beans, soybeans, and corn [47,48]. However, these are annual crops, and the interaction between the fungi and the root of the plants is limited to a shorter period, compared to a perennial crop such as coffee. Thus, the results presented here are novel and promising for application in coffee crops. Applications of entomopathogenic fungi have potential in high-value crops such as coffee and can be of great value to seedling producers, helping the plants acquire protection even in the nurseries, reducing the costs of pesticides, and additionally promoting plant growth. Studies on the persistence of this interaction over time, the need to re-inoculate the fungus in the plant throughout the cycle, and its effects on other key pests of the coffee crop deserve to be studied to optimize the pest management plan with these organisms. 

Another point to be emphasized was the influence of fungal isolates in the reduction in egg production by CLM females, which directly affected the number of individuals from these insects. The mechanisms involved in this effect, however, need to be better elucidated to understand the long-term effect of *Metarhizium* on pest control.

## 5. Conclusions

It is reasonable to conclude that the inoculation of entomopathogenic fungi of the genus *Metarhizium* in coffee seeds may be a promising strategy for not only the management of coffee leaf miners; apparently, its association with plant roots helps reduce the CLM population, increases its time of development, and also increases the development of coffee plants.

## Data Availability

The original contributions presented in the study are included in the article/Supplementary Materials, further inquiries can be directed to the corresponding authors.

## Supplementary Materials

The following supporting information can be downloaded at: https://www.mdpi.com/article/10.3390/microorganisms12091845/s1, Table S1: Comparison between treatments with and without fungicide applied to coffee seeds and their effects on *Leucoptera coffeella* life cicle; Table S2: Comparison between treatments with and without fungicide applied to coffee seeds and their effects on the survival of *Leucoptera coffeella* males; Table S3: Comparison between treatments with and without fungicide applied to coffee seeds and their effects on the survival of *Leucoptera coffeella* females; Figure S1: Reproductive structures of *Metarhizium* fungi recovered from coffee plant roots under a 40× microscope. Here, we can see the group characteristic conidia structure.

## References

[1] Iwanicki N.S., Pereira A.A., Botelho A.B.R.Z., Rezende J.M., de Andrade Moral R., Zucchi M.I., Delalibera Júnior I. (2019). Monitoring of the field application of *Metarhizium anisopliae* in Brazil revealed high molecular diversity of *Metarhizium* spp. in insects, soil and sugarcane roots. Sci. Rep..

[2] Maina U., Galadima I., Gambo F., Dauda Z. (2018). A review on the use of entomopathogenic fungi in the management of insect pests of field crops. J. Entomol. Zool. Stud..

[3] Zimmermann G. (2007). Review on safety of the entomopathogenic fungus *Metarhizium anisopliae*. Biocontrol Sci. Technol..

[4] Li Z., Alves S.B., Roberts D.W., Fan M., Delalibera I., Tang J., Lopes R.B., Faria M., Rangel D.E.N. (2010). Biological control of insects in Brazil and China: History, current programs and reasons for their successes using entomopathogenic fungi. Biocontrol Sci. Technol..

[5] Méndez-González F., Castillo-Minjarez J.M., Loera O., Favela-Torres E. (2022). Current developments in the resistance, quality, and production of entomopathogenic fungi. World J. Microbiol. Biotechnol..

[6] Domingues M.M., Santos P.L., Gêa B.C.C., Carvalho V.R., Oliveira F.N., Soliman E.P., Silva W.M., Zanuncio J.C., Santos Junior V.C., Wilcken C.F. (2022). Isolation and molecular characterization of *Cordyceps* sp. from *Bemisia tabaci* (Hemiptera: Aleyrodidae) and pathogenic to *Glycaspis brimblecombei* (Hemiptera: Aphalaridae). Braz. J. Biol..

[7] Furuie J.L., Stuart A.K.d.C., Voidaleski M.F., Zawadneak M.A.C., Pimentel I.C. (2022). Isolation of *Beauveria* strains and their potential as control agents for *Lema bilineata* Germar (Coleoptera: Chrysomelidae). Insects.

[8] Clifton E.H., Jaronski S.T., Coates B.S., Hodgson E.W., Gassmann A.J. (2018). Effects of endophytic entomopathogenic fungi on soybean aphid and identification of *Metarhizium* isolates from agricultural fields. PLoS ONE.

[9] Franzin M.L., Moreira C.C., da Silva L.N.P., Martins E.F., Fadini M.A.M., Pallini A., Elliot S.L., Venzon M. (2022). *Metarhizium* Associated with coffee seedling roots: Positive effects on plant growth and protection against *Leucoptera coffeella*. Agriculture.

[10] Kabaluk J.T., Ericsson J.D. (2007). *Metarhizium anisopliae* seed treatment increases yield of field corn when applied for wireworm control. Agron. J..

[11] Gonçalves V.P., de Farias C.R.J., Moreira-Nunêz V., Moccellin R., Gaviria-Hernández V., da Rosa A.P.S.A. (2019). Effect of agrochemicals used in the cultivation of soybean and irrigated rice on *Beauveria bassiana* (Bals.) Vuill. and *Metarhizium anisopliae* (Metsch.) Sorok. J. Agric. Sci..

[12] Joshi M., Gaur N., Pandey R. (2018). Compatibility of entomopathogenic fungi *Beauveria bassiana* and *Metarhizium anisopliae* with selective pesticides. J. Entomol. Zool. Stud..

[13] Peng G., Xie J., Guo R., Keyhani N.O., Zeng D., Yang P., Xia Y. (2021). Long-term field evaluation and large-scale application of a *Metarhizium anisopliae* strain for controlling major rice pests. J. Pest Sci..

[14] Presa-Parra E., Hernández-Rosas F., Bernal J.S., Valenzuela-González J.E., Martínez-Tlapa J., Birke A. (2021). Impact of *Metarhizium robertsii* on adults of the parasitoid *Diachasmimorpha longicaudata* and parasitized *Anastrepha ludens* larvae. Insects.

[15] Ahmad I., Jiménez-Gasco M.d.M., Luthe D.S., Shakeel S.N., Barbercheck M.E. (2020). Endophytic *Metarhizium robertsii* promotes maize growth, suppresses insect growth, and alters plant defense gene expression. Biol. Control.

[16] Sheng H., McNamara P.J., St. Leger R.J. (2022). Metarhizium: An opportunistic middleman for multitrophic lifestyles. Curr. Opin. Microbiol..

[17] St. Leger R.J., Wang J.B. (2020). Metarhizium: Jack of all trades, master of many. Open Biol..

[18] Stone L.B.L., Bidochka M.J. (2020). The multifunctional lifestyles of *Metarhizium*: Evolution and applications. Appl. Microbiol. Biotechnol..

[19] Hu S., Bidochka M.J. (2021). Abscisic acid implicated in differential plant responses of Phaseolus vulgaris during endophytic colonization by *Metarhizium* and pathogenic colonization by Fusarium. Sci. Rep..

[20] Rivas-Franco F., Hampton J.G., Narciso J., Rostás M., Wessman P., Saville D.J., Jackson T.A., Glare T.R. (2020). Effects of a maize root pest and fungal pathogen on entomopathogenic fungal rhizosphere colonization, endophytism and induction of plant hormones. Biol. Control.

[21] El-Husseini M.M., Agamy E.A., Bekheit H.K., Ali S.S. (2010). Virulence of Destruxins from two *Metarhizium anisopliae* (Metch.) Isolates versus larvae of the sugar beet worm, *Spodoptera exigua* (Hübner). Egypt. J. Biol. Pest Control.

[22] Golo P.S., Gardner D.R., Grilley M.M., Takemoto J.Y., Krasnoff S.B., Pires M.S., Fernandes É.K.K., Bittencourt V.R.E.P., Roberts D.W. (2014). Production of Destruxins from *Metarhizium* spp. Fungi in artificial medium and endophytically colonized cowpea plants. PLoS ONE.

[23] Reis P.R., Souza J.C., Venzon M. (2002). Manejo ecológico das principais pragas do cafeeiro. Inf. Agropecu.

[24] Reis P.R., de Souza J.C. (1996). Manejo integrado do bicho-mineiro, *Perileucoptera coffeella* (Guérin-Meneville) (Lepidoptera: Lyonetiidae), e seu reflexo na produção de café. An. Soc. Entomológica Bras..

[25] Guedes R.N.C., Walse S.S., Throne J.E. (2017). Sublethal exposure, insecticide resistance, and community stress. Curr. Opin. Insect Sci..

[26] Leite S.A., Dos Santos M.P., Resende-Silva G.A., da Costa D.R., Moreira A.A., Lemos O.L., Guedes R.N.C., Castellani M.A. (2020). Area-wide survey of chlorantraniliprole resistance and control failure likelihood of the Neotropical coffee leaf miner *Leucoptera coffeella* (Lepidoptera: Lyonetiidae). J. Econ. Entomol..

[27] Leite S.A., Santos M.P., Costa D.R., Moreira A.A., Guedes R.N.C., Castellani M.A. (2021). Time concentration interplay in insecticide resistance among populations of the Neotropical coffee leaf miner, *Leucoptera coffeella*. Agric. For. Entomol..

[28] Venzon M. (2021). Agro-ecological management of coffee pests in Brazil. Front. Sustain. Food Syst..

[29] Moreira S.D., França A.C., Rocha W.W., Tibães E.S.R., Neiva Júnior E. (2018). Inoculation with mycorrhizal fungi on the growth and tolerance to water deficit of coffee plants. Rev. Bras. Eng. Agrícola Ambient..

[30] Petch T. (1935). Notes on entomogenous fungi. Trans. Br. Mycol. Soc..

[31] Bischoff J.F., Rehner S.A., Humber R.A. (2009). A multilocus phylogeny of the *Metarhizium* anisopliae lineage. Mycologia.

[32] Canassa F., Tall S., Moral R.A., de Lara I.A., Delalibera I., Meyling N.V. (2019). Effects of bean seed treatment by the entomopathogenic fungi *Metarhizium robertsii* and *Beauveria bassiana* on plant growth, spider mite populations and behavior of predatory mites. Biol. Control.

[33] Martins E.F., Franzin M.L., Perez A.L., Schmidt J.M., Venzon M. (2021). Is *Ceraeochrysa cubana* a coffee leaf miner predator?. Biol. Control.

[34] Lenth R.V., Buerkner P., Herve M., Love J., Riebl H., Singmann H. (2021). Packpage “Emmeans”. https://cran.r-project.org/web/packages/emmeans/index.html.

[35] R CoreTeam (2020). R: A Language and Environment for Statistical Computing.

[36] Liu B.L., Tzeng Y.M. (2012). Development and applications of destruxins: A review. Biotechnol. Adv..

[37] Giuliano G.D.B., Krasnoff S.B., Sun-Moon Y., Churchill A.C.L., Gibson D.M. (2012). Genetic basis of destruxin production in the entomopathogen *Metarhizium robertsii*. Curr. Genet..

[38] Ríos-Moreno A., Garrido-Jurado I., Resquín-Romero G., Arroyo-Manzanares N., Arce L., Quesada-Moraga E. (2016). Destruxin A production by *Metarhizium brunneum* strains during transient endophytic colonization of *Solanum tuberosum*. Biocontrol Sci. Technol..

[39] Gouda S., Das G., Sen S.K., Shin H.-S., Patra J.K. (2016). Endophytes: A treasure house of bioactive compounds of medicinal importance. Front. Microbiol..

[40] Clark R.Á., Zeto S.K. (2000). Mineral acquisition by arbuscular mycorrhizal plants. J. Plant Nutr..

[41] Schenkel D., Maciá-Vicente J.G., Bissell A., Splivallo R. (2018). Fungi indirectly affect plant root architecture by modulating soil volatile organic compounds. Front. Microbiol..

[42] Ahmad I., Jiménez-Gasco M.d.M., Luthe D.S., Barbercheck M.E. (2022). Endophytic *Metarhizium robertsii* suppresses the phytopathogen, *Cochliobolus heterostrophus* and modulates maize defenses. PLoS ONE.

[43] Sasan R.K., Bidochka M.J. (2012). The insect-pathogenic fungus *Metarhizium robertsii* (Clavicipitaceae) is also an endophyte that stimulates plant root development. Am. J. Bot..

[44] MAPA Ministério da Agricultura Pecúária e Abastecimento. https://agrofit.agricultura.gov.br/agrofit_cons/principal_agrofit_cons.

[45] Pava-Ripoll M., Angelini C., Fang W., Wang S., Posada F.J., St. Leger R. (2011). The rhizosphere-competent entomopathogen *Metarhizium anisopliae* expresses a specific subset of genes in plant root exudate. Microbiology.

[46] St. Leger R.J. (2008). Studies on adaptations of *Metarhizium anisopliae* to life in the soil. J. Invertebr. Pathol..

[47] Behie S.W., Zelisko P.M., Bidochka M.J. (2012). Endophytic insect-parasitic fungi translocate nitrogen directly from insects to plants. Science.

[48] Lahey S., Angelone S., DeBartolo M.O., Coutinho-Rodrigues C., Bidochka M.J. (2020). Localization of the insect pathogenic fungal plant symbionts *Metarhizium robertsii* and *Metarhizium brunneum* in bean and corn roots. Fungal Biol..

