# Supplementary material for: Metarhizium-Inoculated Coffee Seeds Promote Plant Growth and Biocontrol of Coffee Leaf Miner
Source: Microorganisms. 2024 Sep 6;12(9):1845. doi: 10.3390/microorganisms12091845 (PMC11433645; doi:10.3390/microorganisms12091845)
Supplement: Supplementary file 1 [file microorganisms-12-01845-s001.zip › microorganisms-3163212-supplementary.pdf]

Supplementary data:

Table S1: Comparison between treatments with and without fungicide applied to coffee seeds and their effects on *Leucoptera coffeella* life cicle

Pairwise comparison	Z value	P value
Control 1 vs Control 2	-13.589	<0.001
Control 1 vs Treatment 1	-18.611	<0.001
Control 1 vs Treatment 2	-20.846	<0.001
Control 1 vs Treatment 3	-17.738	<0.001
Control 1 vs Treatment 4	-23.568	<0.001
Control 2 vs Treatment 1	-11.351	<0.001
Control 2 vs Treatment 2	-12.338	<0.001
Control 2 vs Treatment 3	-10.398	<0.001
Control 2 vs Treatment 4	-14.472	<0.001
Treatment 1 vs Treatment 2	1.192	0.8406
Treatment 1 vs Treatment 3	0.691	0.9828
Treatment 1 vs Treatment 4	0.970	0.9271
Treatment 2 vs Treatment 3	-0.417	0.9984
Treatment 2 vs Treatment 4	-0.345	0.9994
Treatment 3 vs Treatment 4	0.150	1.0000

Table S2: Comparison between treatments with and without fungicide applied to coffee seeds and their effects on the survival of *Leucoptera coffeella* males

Pairwise comparison	Z value	P value
Control 1 vs Control 2	0.238	0.9999
Control 1 vs Treatment 1	0.038	1.0000
Control 1 vs Treatment 2	0.323	0.9995
Control 1 vs Treatment 3	0.592	0.9913
Control 1 vs Treatment 4	0.545	0.9941
Control 2 vs Treatment 1	-0.200	1.0000
Control 2 vs Treatment 2	0.084	1.0000
Control 2 vs Treatment 3	0.354	0.9992
Control 2 vs Treatment 4	0.307	0.9996
Treatment 1 vs Treatment 2	0.284	0.9997
Treatment 1 vs Treatment 3	0.554	0.9936
Treatment 1 vs Treatment 4	0.507	0.9958
Treatment 2 vs Treatment 3	0.270	0.9998
Treatment 2 vs Treatment 4	0.223	0.9999
Treatment 3 vs Treatment 4	-0.047	1.0000

Table S3: Comparison between treatments with and without fungicide applied to coffee seeds and their effects on the survival of *Leucoptera coffeella* females

Pairwise comparison	Z value	P value
Control 1 vs Control 2	-0.302	0.9997
Control 1 vs Treatment 1	0.445	0.9977
Control 1 vs Treatment 2	-0.343	0.9994
Control 1 vs Treatment 3	0.392	0.9988
Control 1 vs Treatment 4	0.392	0.9978
Control 2 vs Treatment 1	0.747	0.9753
Control 2 vs Treatment 2	-0.041	1.0000
Control 2 vs Treatment 3	0.694	0.9821
Control 2 vs Treatment 4	0.694	0.9721
Treatment 1 vs Treatment 2	-0.788	0.9689
Treatment 1 vs Treatment 3	-0.053	1.0000
Treatment 1 vs Treatment 4	-0.053	1.0000
Treatment 2 vs Treatment 3	0.735	0.9770
Treatment 2 vs Treatment 4	0.735	0.9510
Treatment 3 vs Treatment 4	-0.051	0.9320

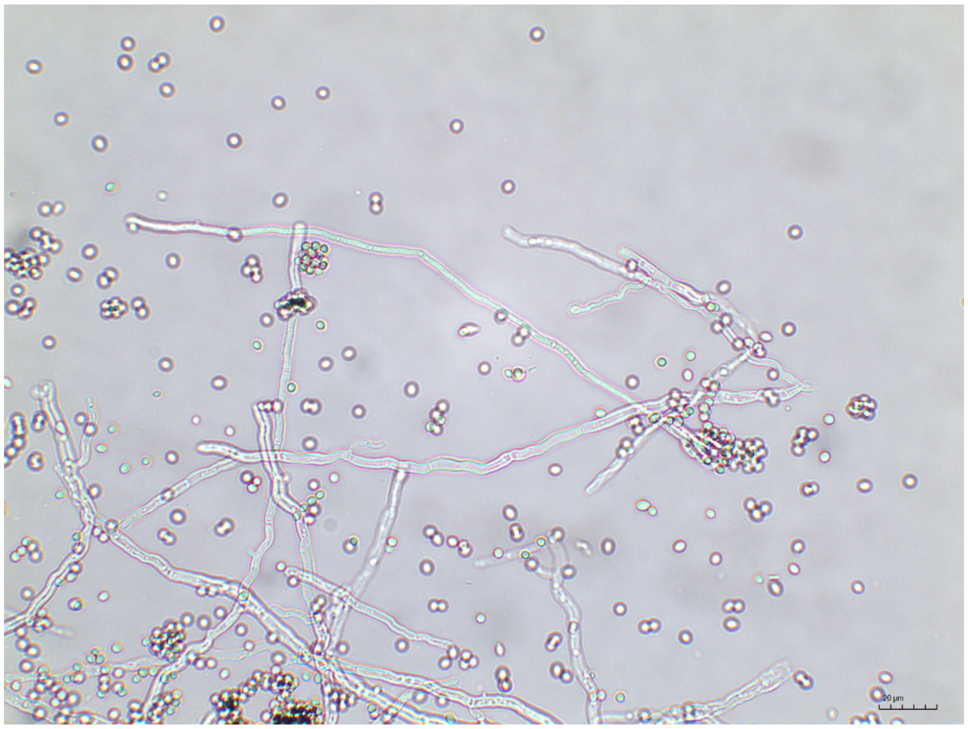

Figure S1: Reproductive structures of *Metarhizium* fungi recovered from coffee plant roots under a 40x microscope. Here, we can see the group characteristic conidia structure.
